# NK Cells, Tumor Cell Transition, and Tumor Progression in Solid Malignancies: New Hints for NK-Based Immunotherapy?

**DOI:** 10.1155/2016/4684268

**Published:** 2016-05-12

**Authors:** Claudia Cantoni, Leticia Huergo-Zapico, Monica Parodi, Marco Pedrazzi, Maria Cristina Mingari, Alessandro Moretta, Bianca Sparatore, Segundo Gonzalez, Daniel Olive, Cristina Bottino, Roberta Castriconi, Massimo Vitale

**Affiliations:** ^1^Department of Experimental Medicine (DIMES), University of Genoa, 16132 Genova, Italy; ^2^Center of Excellence for Biomedical Research (CEBR), University of Genoa, 16132 Genova, Italy; ^3^Istituto Giannina Gaslini, 16147 Genova, Italy; ^4^IRCCS AOU San Martino-IST, 16132 Genova, Italy; ^5^Department of Functional Biology, IUOPA, University of Oviedo, 33006 Oviedo, Spain; ^6^U1068, CRCM, Immunity and Cancer, INSERM, 1312 Marseille, France

## Abstract

Several evidences suggest that NK cells can patrol the body and eliminate tumors in their initial phases but may hardly control established solid tumors. Multiple factors, including the transition of tumor cells towards a proinvasive/prometastatic phenotype, the immunosuppressive effect of the tumor microenvironment, and the tumor structure complexity, may account for limited NK cell efficacy. Several putative mechanisms of NK cell suppression have been defined in these last years; conversely, the cross talk between NK cells and tumor cells undergoing different transitional phases remains poorly explored. Nevertheless, recent* in vitro* studies and immunohistochemical analyses on tumor biopsies suggest that NK cells could not only kill tumor cells but also influence their evolution. Indeed, NK cells may induce tumor cells to change the expression of HLA-I, PD-L1, or NKG2D-L and modulate their susceptibility to the immune response. Moreover, NK cells may be preferentially located in the borders of tumor masses, where, indeed, tumor cells can undergo Epithelial-to-Mesenchymal Transition (EMT) acquiring prometastatic phenotype. Finally, the recently highlighted role of HMGB1 both in EMT and in amplifying the recruitment of NK cells provides further hints on a possible effect of NK cells on tumor progression and fosters new studies on this issue.

## 1. Introduction

NK cells are Innate Lymphoid Cells (ILCs) that play a crucial role in the defense against viruses and in the surveillance of tumor insurgence [1–5]. In view of their possible exploitation in cancer (but also in viral infections), these cells have been intensively studied, so that the molecular mechanisms regulating their antitumor cytolytic activity have been extensively defined. By the use of a wide array of surface receptors capable of delivering either triggering or inhibitory signals, NK cells can monitor surrounding cells, checking for their possible phenotypic alterations, and tune an appropriate cytolytic response. In humans, these receptors are essentially represented by the following: (1) the HLA-I-specific inhibitory receptors, Killer Ig-like Receptors (KIR), and CD94:NKG2A receptor, which prevent NK cells from attacking normal autologous cells, and (2) a number of activating receptors (including NKG2D, DNAM-1, and the Natural Cytotoxicity Receptors (NCRs): NKp46, NKp30, and NKp44), which recognize ligands expressed on the surface of transformed and/or virally infected cells and enable NK cells to kill abnormal cells [3, 6].

Most of the above-mentioned receptors are also involved in the control of additional functions exerted by NK cells ranging from the release of cytokines and chemokines (namely, IFN-*γ*, TNF-*α*, GM-CSF, MIP1-*α*, and RANTES) [3, 7] to the regulatory interactions with different immune cell types including Dendritic Cells (DCs), macrophages, monocytes, granulocytes, and T cells [3, 8–12]. Moreover, NK cells are endowed with additional diverse receptors that enable them to respond to a variegated plethora of stimuli. Thus, NK cells can variably potentiate their functions in response to several Pathogen Associated Molecular Patterns (PAMPs) by using different TLRs (i.e., TLR2, TLR3, TLR7, and TLR9) [13, 14]; they can strongly increase their cytokine production and/or their cytolytic properties in response to different cytokines including IL-2, IL-15, IL-12, IL-18, and IFNs *α*/*β* [3, 15]; and finally, they can also migrate in response to various chemotactic stimuli (see below). Two main NK cell functions (i.e., cytotoxicity and IFN-*γ* production) appear to be differently distributed among specific NK cell subsets in Peripheral Blood (PB) and Lymph Nodes (LN). The so-called “terminally differentiated” PB CD56^dim^CD16^bright^ NK cells expressing CD57 and KIR molecules display a high cytotoxic potential and a limited ability to secrete IFN-*γ* upon cytokine stimulation. The CD56^dim^CD16^bright^CD57^−^KIR^−^NKG2A^+^ PB NK cells exert both functions at intermediate levels. Finally, less differentiated CD56^bright^CD16^dim/^ ^neg^CD57^−^KIR^−^NKG2A^++^ NK cells, which preferentially locate in LN and are poorly represented in PB, show low cytotoxicity and high IFN-*γ* release upon cytokine stimulation [15–18]. Remarkably, it has been also proposed that NK cells may adapt their cytolytic potential to the pattern of NK receptor ligands (NKR-Ls) stably expressed in the milieu. Thus, the chronic exposure to activating ligands or to abnormally low levels of MHC-I molecules (i.e., inhibitory ligands) would render NK cells poorly reactive. On the other hand, the exposure to adequate MHC-I levels would increase NK cell reactivity and would be essential for differentiated KIR^+^ NK cells to become fully competent [19].

This brief description of the NK cell biology indicates that these cells are far from being simple cytolytic effectors capable of killing different tumor cell targets; rather, they represent a heterogeneous population that is able to fulfill different functions and to finely tune its activity in variable environmental contexts. Such emerging complexity renders the exploitation of NK cells for effective immunotherapies more complicated than initially thought, especially in the context of solid tumors. Indeed, while different animal models and a follow-up study support the notion that NK cells can survey and control the insurgence of tumors [20–22], a straightforward role of NK cells in the control of advanced established solid tumors is far from being defined. In this context the specific tumor associated microenvironment evolving along with the progression of the malignancy may play a role. On the one hand, the increasing tumor structure complexity and the tumor microenvironment can heavily affect NK cell behavior and limit NK cell infiltration of the tumor mass; on the other hand, NK cells that successfully reach (or develop within) the tumor site may interact with different cell populations and influence the progression of the tumor. In the past few years, the suppressive effects of the tumor microenvironment on NK cells have been widely studied [23, 24]. A number of soluble factors [25–28], as well as different tumor associated regulatory/suppressive immune cells [29, 30], Tumor Associated Fibroblasts (TAFs) [31], and tumor cells [32, 33], have been shown to profoundly alter the expression and/or the function of several NK cell receptors and affect the ability of NK cells to reach, recognize, and kill tumor cells at the tumor site (Table 1). Conversely, the possible effect of NK cells on tumor progression is still poorly investigated. In this context, recent evidences indicate that NK cells are capable of modifying the immunogenicity of cancer cells (see below).

The structure of tumor tissue is rather complex and encompasses hypoxic niches, vascularized areas, necrotic zones, and a front of tumor invasion. Along this front, several tumor cells can acquire a less differentiated prometastatic phenotype through a transitional process that in tumors of epithelial origin is referred to as Epithelial-to-Mesenchymal Transition (EMT) (or EMT-like process in melanomas) [48–50]. Thus, the positioning of NK cells within the tumor and their possible conditioning in a hypoxic environment [51] represent important additional elements to be considered in order to define the role of NK cells in the progression of tumor. In this context High Mobility Group Box-1 (HMGB1) [52] may represent a link between NK cells and tumor cell progression. Indeed, recent studies have provided evidence for a role of HMGB1 in the induction of the EMT [53], while in our lab we have recently shown that HMGB1 is actively released during NK:melanoma cell interaction and can amplify recruitment of NK cells.

In this review we analyze the current information suggesting a possible role for infiltrating NK cells in the evolution of tumor cells towards more malignant stages. In this context we also discuss HMGB1 as a possible key-player linking NK cells to the plasticity of tumor cells.

## 2. NK Cells and the Progression of Cell Tumorigenesis

### 2.1. The Process of Tumorigenesis and Tumor Cell Plasticity

Normal cell growth and death are tightly controlled processes that ensure the maintenance of tissue homeostasis in the body. The occurrence of random mutations affecting key suppressor genes, oncogenes, and genes involved in DNA repair can alter such homeostatic status, leading to uncontrolled cell growth, immortality, and, finally, tumor onset. In solid tumors the specific microenvironment may enhance the genetic and epigenetic instability of evolving tumor cells, favoring the accumulation of mutations and the progression towards a proinvasive and prometastatic phenotype. Epigenetic changes may contribute to late evolution steps by conferring phenotypic and functional plasticity to tumor cells [48, 54]. The acquisition of plasticity is relevant to the capacity of tumor cells to leave the primary tumor, spread to distant organs through the blood stream or the lymphatic system, and create metastases [55]. In cancers of epithelial origin, tumor cells must lose their epithelial characteristics (polarity, cell-to-cell adhesion, etc.) and gain mesenchymal traits that allow them to detach from the primary site and invade both neighboring and distant tissues. Acquisition of migrating and invasive properties, as well as turning back into an epithelial phenotype to establish micrometastases, implies a vast cell reprogramming that borrows the molecular pathways from the latent developmental program known as Epithelial-to-Mesenchymal Transition/Mesenchymal-to-Epithelial Transition (EMT/MET) [48].

### 2.2. The EMT and Its Possible Relationship with the Immune System

The induction of EMT seems to be rather tissue-specific and is governed by complex networks. TGF-*β*-, Wnt-, Notch-, and growth factor receptor-induced signaling cascades are the main inducers of the EMT; not coincidentally, most of these pathways are overactivated in carcinomas and have been associated with the acquisition of an invasive phenotype [55, 56]. In addition, recent studies have demonstrated that adverse cellular conditions, such as hypoxia or some components of the extracellular matrix (i.e., collagen and hyaluronic acid), can also induce EMT in some cancer types [57–59].

Despite their complexity, the EMT-inducing pathways have a common endpoint in the activation of a short list of EMT-inducing transcription factors (TF) (including Snail, Slug, zinc finger E-box binding homeobox 1 (ZEB1), Twist, Goosecoid, and FOXC2) and MET-inducing microRNAs (miR200 and miR34) that orchestrate the phenotype switch [60]. The consequence of the activation of all these TF is the transcriptional repression of cellular junction proteins, such as E-cadherin, claudins, and ZO-1, causing the loss of epithelial integrity and also the activation of programs associated with tumor invasion [61–65]. Instead, noncoding microRNAs, microRNA 200 (miR200) and miR205, operate the MET process inhibiting the repressors of E-cadherin expression, ZEB1 and ZEB2, and thereby maintaining the epithelial cell phenotype [66, 67].

Importantly, the recent advances in the characterization of tumor cell plasticity and phenotype switching on epithelial and nonepithelial tumors (i.e., melanomas) suggest that the EMT-MET could be part of a wider, complex transitional phenomenon, in which tumor cells may fluctuate from a differentiated, proliferative, poorly invasive, drug sensitive phenotype to an undifferentiated, poorly proliferative, proinvasive, drug resistant phenotype [68–71]. Thus, tumor cell plasticity and its regulation appear to be a nodal point in the tumor progression and spread; consequently, targeting this phenomenon may be crucial for the immune system to control the fate of the tumor.

Immune cells may have a dual role in carcinogenesis [71, 72]. While a powerful antitumor immune response often occurs to control the first steps of malignant transformation [73–75], in the later tumor stages, transformed cells may manage not only to counteract the immune function but also to use it as an ally in tumor progression [76]. In this context, recent studies have shown that EMT could be locally associated with the presence of granulocytes, Tregs, M2 macrophages, or MDSC, indicating a role for different immune cell types in EMT induction [77–82]. These studies also suggest that EMT may preferentially induce or be induced by suppressive immune cells. Moreover, EMT may directly confer mesenchymal-like immune-modulatory features to tumor cells [83]. Finally, EMT may also modify the immunogenicity of cancer cells, favoring their escape from the T cell-mediated attack (immunoediting) [72, 84, 85]. A link between immunoediting and EMT promotion has been investigated in syngeneic immune-competent mice transplanted with epithelial cancers expressing the neu*-*oncogene [84]. As expected, the immune surveillance resulted in a macroscopic elimination of the tumor that however was not complete. Indeed, tumor relapse occurred and the new lesions were enriched in neu-negative malignant cells with a mesenchymal phenotype.

Given the potential antitumor and antimetastatic effect of NK cells, an important (still open) question is whether and how NK cells could functionally interact with cells undergoing EMT and how relevant this potential interaction could be to the control of the malignancy.

### 2.3. NK Cells and EMT

The mutual influence between EMT and NK cells has been barely investigated until now. Only few studies address the issue of the potential changes in tumor immunogenicity during EMT and its implications for NK cell-mediated responses.

In a recent study, López-Soto and colleagues described a significant upregulation of NKG2D activating ligands in colon cancer cells undergoing EMT, as well as a remarkable downregulation of HLA-I expression. They proposed that the EMT could enhance cancer cell immunogenicity towards NK cells and favor tumor clearance in a NKG2D-dependent manner. They also showed that the expression of MICA/B proteins (the main ligands for the NKG2D receptor) was very low in advanced* in vivo* tumors with invasive properties. Concomitantly, a greater presence of NKG2D^+^ cytotoxic Tumor-Infiltrating Lymphocytes (TILs) was observed in these samples. These results suggested that NK cells, through the engagement of NKG2D receptor, might be responsible for the elimination of MICA/B-expressing transitional cells, thus exerting an immunoediting of tumor cells by the selection of less immunogenic variants [86, 87]. In line with these data, Chen and colleagues showed that prostate cancer cells strongly downregulate HLA-I expression during TGF-*β*/EGF-induced EMT in a Snail-dependent manner. This phenotypic change might render tumor cells resistant to cytotoxic T cell-mediated lysis but might also increase their susceptibility to NK cell-mediated responses. On the other hand, TGF-*β*, besides inducing EMT, could also suppress NK cells and compensate the effect of the decreased resistance to NK cells acquired by transitional tumor cells [88].

Compelling evidences indicate that the invasive behavior of tumor cells may be strongly influenced by nearby stromal and immune cells. Nonetheless, regarding the possible effect of NK cells on the induction or inhibition of EMT there are presently no direct data. However, different studies indicated that NK cells can modulate the phenotype of tumor cells and modify their immunogenicity to either NK or T cells. We have recently shown that NK cells can induce HLA-I upregulation on the surface of melanoma cells and confer resistance to NK cell-mediated killing [89]. By coculturing melanoma and NK cells at ratios reflecting the level of NK cell infiltrates observed at the tumor site, we described that the initial tumor cell killing was followed by an equilibrium phase characterized by the upregulation on melanoma cells of both classical and nonclassical HLA-I molecules. This effect was mediated by IFN-*γ*, which was released by NK cells upon melanoma cell recognition. This NK cell-mediated immunoediting recalls the so-called “adaptive immune resistance,” a process in which cancer cells adapt their phenotype under the pressure of the immune response in order to evade it. This phenomenon was hypothesized by Taube and coworkers to describe the acquisition of the inhibitory ligand PD-L1 by tumor cells. In particular, this study showed a clear association between the presence of TILs and IFN-*γ* close to tumor cells and PD-L1 expression [90]. Thus, the activation of TILs and the consequent release of IFN-*γ* (but also the expression of IL-10 and IL-32-gamma in the tumor tissue) would result in upregulation of PD-L1 expression [91, 92]. As IFN-*γ* producers, NK cells might significantly enhance PD-L1 expression on tumor cells. In this context it has been recently shown that supernatants conditioned by IL-2-activated NK cells could increase PD-L1 expression on hematopoietic tumor cell lines and primary Multiple Myeloma (MM), Acute Myeloid Leukemia (AML), and Acute Lymphoblastic Leukemia (ALL) cancer cells [93]. Interestingly, in lung cancer, a recent report showed an important correlation between PD-L1 expression and EMT score [94].

A role for NK cells in the modulation of tumor cell phenotype may be particularly significant in the context of tumor cells that express very low levels of HLA-I and cannot efficiently stimulate T cells. Neuroblastoma cells often show low or negative HLA-I surface expression [95, 96]; in this case, the adaptive immune resistance/immunoediting may be driven by NK cells rather than T cells. However, in spite of the lack of HLA-I expression, neuroblasts isolated from bone marrow aspirates have been shown to be quite resistant to NK cell-mediated killing. This resistance was associated with the lack of ligands for activating receptors (i.e., PVR, recognized by DNAM-1) or with the high expression of B7-H3, a ligand for a still unknown inhibitory NK receptor [96]. Thus, these metastatic neuroblasts may hardly stimulate both T and NK cells, which may result in a lack of IFN-*γ* production. This situation may account for the recent observation that such aggressive neuroblasts do not constitutively express PD-L1 [97]. Along this line, it has been recently shown that, indeed, metastatic neuroblasts can significantly acquire PD-L1 (and HLA-I) expression in response to IFN-*γ* [97].

To conclude this issue, it should be considered that an actual evaluation of the possible effects of NK cells on the tumor cell phenotype and plasticity cannot disregard the effective location of the NK infiltrate in the tumor tissue.

## 3. Infiltration of NK Cells in Solid Tumors

The study of the NK cell infiltrate in solid tumors has been made possible only recently, thanks to the generation of new reagents for the specific detection of NK cells by immunohistochemistry and the availability of even more efficient approaches to isolate and/or analyze specific lymphocyte populations from tissues.

The NK cell infiltrate has been assessed in several types of solid tumors including melanomas [89, 98], GastroIntestinal Stromal Tumors (GIST) [47], and colorectal [99], renal [44], lung [100], and breast cancers [45] (Table 1).

In some cases a role for infiltrating NK cells in the control of tumor progression could be also inferred. Two studies on renal carcinoma lung metastases and GIST found a correlation between the levels of the NK infiltrate and better prognosis [37, 44]. Another interesting study on GIST showed that the number of NKp46^+^ TILs inversely correlated with the presence of metastases at diagnosis and indicated that different isoforms of the NKp30 activating receptor could associate with reduced or prolonged survival of the patients [46]. Ali and colleagues have also recently shown that tumor-infiltrated LN of melanoma patients are enriched in highly cytotoxic CD56^dim^CD57^+^KIR^+^ NK cells (instead of the poorly cytotoxic CD56^bright^ NK cells which are typically located in LN) [41]. In this context, it should be considered that the cytotoxic activity of the NK cell pool can significantly vary among individuals and that low PB NK cell activity has been associated in the past with an increased risk of many cancers [22, 101].

On the other hand, several data available in the literature call into question the real effect of NK cells on the progression of the tumor. Some studies indicate a scarce or moderate NK cell infiltration in melanomas, colon cancers, and tumor tissues microarray [42, 89, 102]. In addition, studies addressing the phenotype and function of tumor-infiltrating NK cells have shown an enrichment of poorly cytotoxic CD56^bright^ NK cells (in lung and breast tumor tissues) or the presence of altered poorly functional CD56^dim^ NK cells in different tumor types (see Table 1). Finally, independent studies on colorectal cancer, melanoma, and GIST have shown that NK cells may be preferentially located in the stroma, rather than in direct contact with tumor cells [23].

Overall, these conflicting data point out the still open question on how NK cell recruitment and migration are specifically regulated within the tumor tissue. As generally conceived, the migration process would depend on cell-to-cell and cell-to-extracellular matrix (ECM) interactions, modulation of ECM components, the presence of specific chemokines, and the pattern of chemokine receptors expressed by various NK cell types [103].

The chemokine receptor patterns of the most studied PB NK cell subsets (i.e., the CD56^bright^ cells and the composite group of the CD56^dim^ cells) have been roughly defined, although some published studies are not concordant with regard to the expression of certain specific receptors. Such discrepancies may depend on the sensitivity of the antibodies used for flow cytometric analysis or on the use of different cell isolation techniques (which may alter the chemokine receptor recognition by the specific reagent) [104, 105].

Well-established data indicate that the CD56^dim^CD16^+^ cells express CXCR1, ChemR23, and CX_3_CR1 at high levels and respond to CXCL8 and CX_3_CL1 [106]. Accordingly, these cells may cross the endothelium and reach inflamed tissues or tumor masses (as chronic inflammation often characterizes tumor microenvironment). Moreover, CD56^dim^CD16^+^ cells also express low levels of CXCR2 and CXCR3 but lack CCR7 and CXCR5. By contrast, CD56^bright^CD16^−^ cells express high levels of CCR7 and CXCR3 suggesting that this cell subset would migrate in response to CCL19, CCL21, CXCL9, CXCL10, and CXCL11. CD56^bright^CD16^−^ cells also express low levels of CX_3_CR1 and lack CXCR1, CXCR2, and CXCR5 [15, 104, 105, 107, 108]. Such chemokine receptor pattern is consistent with the prevalent localization of CD56^bright^ cells in LN, but also with the recent observation that an infiltration of CD56^bright^ cells can be detected in tumors showing high CCL19, CCL21, and CXCL9 transcripts [35].

It should be also considered that NK cells can modify the expression of chemokines and chemokine receptors following cytokine stimulation. For example, short term stimulation of NK cells with IL-2 and/or IL-12 results in a decreased expression of CXCR3 [109], while long term exposure to IL-2 upregulates CCR1, CCR2, CCR4, CCR5, and CCR8 and downregulates the expression of CXCR2 and ChemR23 [110]. In addition, IL-2 can also modulate the expression of CCR7 and induce CCR4 and CX3CR1 expression. IL-15 stimulation causes a decrease of CXCR4 and CX3CR1 expression [111]; IL-18 enhances the response to CCL21 through the induction of CCR7 [112]; TGF-*β* induces CXCR3 and CXCR4 expression while deeply reducing CX_3_CR1 surface levels [25]. Therefore, the composition and the localization of the NK cell infiltrate would greatly vary, depending on the type of cytokines and chemokines available in the tumor. In mice, using NK cell-sensitive tumor models, it has been shown that IFN-*γ* can induce the release of CXCL9-10 by tumor-infiltrating immune cells leading to the recruitment of CXCR3^+^ NK cells [113]. In another study on mice it has been shown that the chemoattractant molecule chemerin can favor the recruitment of NK cells in B16 transplantable melanomas [114]. Interestingly, in humans, the expression of the gene coding for chemerin was downregulated in several tumor types [115]. In a recent study, comparing PB NK cells from healthy donors and neuroblastoma patients it has been shown that patients' CD56^dim^ NK cells display a significantly reduced expression of CX_3_CR1, a chemokine receptor involved in the process of cell extravasation [25]. In another study Halama and colleagues have shown a scarce NK cell infiltrate in colorectal cancers despite the high levels of NK cell-attracting chemokines within the tumor. These findings indicate that tumor-orchestrated escape mechanisms may affect NK cell viability in the tumor niche or inhibit the recruitment of NK cells at the tumor site, but also suggest that, besides chemokines, additional chemoattractant molecules may be necessary for the recruitment [42]. Along this line, the role of HMGB1 as chemoattractant for NK cells in tumors has been recently highlighted (see below). Actually, this pleiotropic molecule has recently come into play in various aspects of the tumor biology, not least EMT.

## 4. HMGB1, Immune Cells, and Tumors

HMGB1 is a widely expressed protein mainly localized in the cell nucleus and involved in chromatin remodeling and transcription [116]. However, following cell activation by various physiopathological stimuli, this protein can undergo posttranslational modifications that promote its translocation to the cytosol and its export outside the cell, via a nonclassic secretion pathway that requires LAMP1 positive lysosomes [117]. HMGB1 can also undergo a passive release from damaged or necrotic cells and behaves as a Damage Associated Molecular Pattern (DAMP) able to trigger and amplify both inflammatory and immune responses [118, 119]. Autophagy induction is required and sufficient to cause the release of HMGB1 from dying cells, suggesting that manipulation of autophagy during cancer treatment may influence the immunogenicity of dying tumor cells [120]. Furthermore, HMGB1 has been identified as a cytokine-releasing factor that stimulates the production of TNF-*α*, IL-1*β*, IL-6, and IL-8 from macrophages and neutrophils [121].

The signaling properties of HMGB1 are influenced by the redox state of its cysteine residues localized at positions 23, 45, and 106 [122]. At this respect, all-thiol (i.e., fully reduced) HMGB1 interacts with CXCL12 and the heterocomplex behaves as a chemoattractant for mouse macrophages and fibroblasts and for human monocytes by the engagement of CXCR4 [123–125]. Moreover, HMGB1 can also induce chemotaxis of human monocyte-derived immature DCs through the engagement of RAGE [126]. Conversely, a partial and reversible oxidation of HMGB1 (C23-C45 disulfide bond) is required to activate a TLR4-mediated production of cytokines from macrophages [127]. Instead, HMGB1 released from apoptotic cells is characterized by the irreversible oxidation of C106 to sulfonic acid by ROS. In this oxidation state HMGB1 lacks cytokine-inducing activity, induces tolerogenic DCs, and promotes cell death following treatment with chemotherapeutic agents [128, 129]. Finally, a fully oxidized HMGB1 form has been also described, but its functions have not been yet assessed.

In addition to the redox status, several posttranslational modifications may be relevant to HMGB1 function. So far, the identified modifications on HMGB1 molecules exported from innate immune cells consist of hyperacetylation [130, 131], poly-ADP ribosylation [132], and phosphorylation by CAMK IV and PKC [133–136]. The effects of these molecular changes on the affinity of HMGB1 for its different receptors and their role in cell responses in solid tumors have not been yet explored.

Finally, it should also be considered that HMGB1 can associate with various soluble HMGB1-binding molecules, such as IL-1*β*, LPS, Pam(3)Csk(4), and the above-mentioned CXCL12, and enhance their immunostimulatory activity [137, 138].

Due to its molecular plasticity, HMGB1, either alone or complexed with other molecules, can interact with several receptors including RAGE, IL-1R, TLR2, TLR4, CXCR4, NMDA-R, and TIM-3 [137–141].

Several different stimuli are able to trigger the release of HMGB1 from cells of the innate immune system. In particular, an active export of HMGB1 is induced on monocytes, macrophages, and DCs activated by PAMPs, DAMPs, or cytokines. In addition, also NK cells can actively release HMGB1 in the context of the NK:DC cross talk or upon the engagement of different activating NK cell receptors, including those mainly involved in tumor cell recognition [130, 142–144]. On the other hand, neutrophils mostly undergo a passive release of HMGB1 following cell injury/necrosis [145], whereas, to our knowledge, no information on the export of HMGB1 from eosinophils and basophils is available. Besides innate immune cells, endothelial cells and fibroblasts can also actively release HMGB1 following the exposure to uric acid and LPS, respectively [146, 147]. It should be also noted that several innate immune cells are targets of HMGB1. In particular, on monocyte-derived immature DCs, HMGB1 upregulates specific maturation markers (CD80, CD83, CD86, and HLA-I), enhances the production of cytokines (IL-6, CXCL8, IL-12 p70, and TNF-*α*), switches their chemokine responsiveness from CCL5-sensitive to CCL21-sensitive, and induces the cell capacity to stimulate allogeneic T cell proliferation [126]. Moreover, HMGB1 released from primary tumors can reach the regional LN and weaken their antimetastatic capability by lowering the number of resident macrophages [148]. Depending on its concentration, HMGB1 can induce or inhibit neutrophil chemotactic responses by the engagement of RAGE, TLR2, and TLR4 [149]. The essential role of HMGB1 as a crucial modulator of innate immunity in tumors is supported by* in vivo* evidences showing that HMGB1-deficient tumors display an impaired ability to recruit innate immune cells, including macrophages, neutrophils, and NK cells into the tumor tissue following DNA alkylating therapy [150].

The intracellular amount of HMGB1 is significantly increased in several human tumors such as lung [151], bladder [152], colorectal [153], head and neck [154], prostate [155], hepatocellular [156], and gastric cancer [157] and melanoma [158]. This observation suggests that tumor cells can release high amounts of HMGB1 either by membrane leakage or by active release in inflammatory or hypoxic conditions that are often observed in solid tumors and associated with an increased prometastatic behavior [159]. Extracellular HMGB1 can play further roles in cancer through the activation of endothelial cells. Specifically, the all-thiol form displays a proangiogenic activity that supports tumor growth [160, 161]. The heterogeneous responses observed in different conditions could depend on the local concentration of the different redox forms of the protein, on the type of cellular receptors engaged by HMGB1, and on the presence of specific soluble HMGB1-binding molecules [137, 138]. Thus, HMGB1 from both tumor and immune cells can accumulate in the tumor microenvironment and sustain inflammation, cytokine release, cell proliferation, and recruitment of immune cells. The full characterization of these effects and the evaluation of their importance in the context of the tumor development (or control) may be crucial for the identification of new checkpoints in the host:tumor interaction and for the definition of effective therapeutic targets. In this context we have recently described a new mechanism by which HMGB1 could strongly influence the presence and the efficacy of NK cells at the tumor site.

### 4.1. Role of HMGB1 in NK Cell-Tumor Interaction

**Figure 1:**
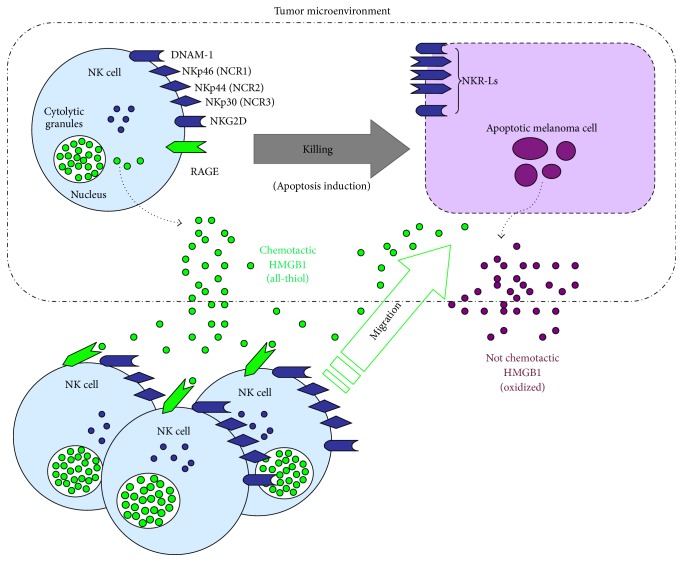
New mechanism proposed for NK cell recruitment at melanoma tumor site. NK cells recognize melanoma cells through the interaction of NK activating receptors with their ligands (NKR-Ls) expressed on tumor cell (both depicted in dark blue). The resulting NK cell activation leads to the killing of melanoma cells (via perforin/granzyme B and induction of apoptosis) and to the active release of a chemotactic form of HMGB1 (green circles). Killed (apoptotic) cells passively release HMGB1 as oxidized molecule. However, this HMGB1 form has no chemotactic properties (purple circles). NK-derived reduced (i.e., all-thiol) HMGB1 can act as chemoattractant for activated NK cells through the engagement of RAGE (depicted in green), thus promoting their recruitment in the tumor microenvironment.

We have recently addressed the role of HMGB1 in the context of the innate immune response against tumors by investigating the function of this protein in the NK:melanoma cell interaction and in the subsequent NK-mediated killing of tumor cells. We have shown that, during the interaction with melanoma cells, NK cells could release an HMGB1 form endowed with chemotactic activity, while killed melanoma cells passively released an oxidized, nonchemotactic form of HMGB1 (Figure 1). The chemotactic HMGB1 could potently attract activated NK cells through the engagement of RAGE, which, indeed, was expressed at the surface of NK cells. Interestingly, after prolonged exposure to HMGB1, NK cells did not enhance their chemotactic properties; rather, they showed an increased cell motility, which was accompanied by expression changes in several proteins involved in the regulation of the cytoskeletal network [144]. Thus, our finding defined a new mechanism by which HMGB1 could sustain the antitumor function of NK cells. Indeed, HMGB1 could initially play a crucial role in amplifying the NK cell recruitment to the site of NK:tumor cell interaction; next, it could improve the patrolling capability of NK cells that have reached the tumor by enhancing their motility.

The presence of HMGB1 in the context of the NK:tumor cell interaction may also play a role in the progression of the tumor. Recent observations show that HMGB1 is a potent driver of EMT in colorectal carcinoma via the activation of the RAGE/Snail/NF-*κ*B pathway and of MMP-7 [53]. NK cells have been shown to be frequently located in the front of invasion of the tumor, where, indeed, the EMT process is likely to occur. Thus, in this situation, HMGB1 may recall additional NK cells in the area, which, in turn, would release further HMGB1 thus contributing to the EMT.

It is worth noting that NK cells also express TIM-3, which has been shown to recognize HMGB1 [162]. Moreover, HMGB1 may also influence NK cell function by its ability to potentiate the activity of the NMDA receptor [141]. Indeed, the activation of this protein channel in human NK cells, T lymphocytes, and neutrophils has been shown to increase the production of ROS [163]. These receptors do not appear to modulate HMGB1-mediated NK chemotaxis, but their possible involvement in additional functions cannot be ruled out.

## 5. Concluding Remarks

While several reports demonstrate the inefficacy of NK cells in controlling tumor growth and invasion, NK cell role in the prevention of metastasis has been described in different types of cancer, and a higher number of tumor-infiltrating NK cells have been associated with a better prognosis [20–24]. Thus, it is not surprising that in the past years these ILCs have starred in cancer immunotherapy clinical trials with promising results. Therefore, it could be unpopular to ask about their potential role in the tumor progression. Nonetheless, during antitumor immune responses, NK cells can represent a source of IFN-*γ*, which potentially promotes the adaptive immune resistance of tumor cells, and TNF-*α*, a known EMT inducer. In addition, NK cells may be often located within the stroma, at the interface with the invasive front of the tumor, where, indeed, the EMT (i.e., the transitional tumor cell phenotype) is frequently observed. Thus several hints foster the idea that NK cells, in spite of their potential ability to control metastases, may also play an unwanted role in the promotion of cancer plasticity. This controversial issue should be definitively clarified. The definition of whether and how NK cells are recruited, migrate within the tumor, and influence the EMT, along with the new insights into the putative role of HMGB1, would provide new important elements to maximize the still unexplored potential of NK cells in the therapy of solid tumors.

## Figures and Tables

**Table 1:** NK cell infiltrate in solid tumors.

Tumor	NK cell infiltrate: phenotype	NK cell infiltrate: size and/or location	Ref.
Lung adenocarcinoma	Reduced expression of NKp30, NKp46		[34]
	Enrichment of CD56^bright^ Perf^low^ poorly cytotoxic NK cells		[35]

Non-small cell lung cancer	CD56^bright^CD16^dim^ infiltrating NK cells with impaired killing capability		[36]
	Infiltrating CD56^dim^ with low activating NK-rec expression and function	NKp46^+^ cells mainly localized at the invasive margin	[37]

Melanoma (primary)		Moderate/low CD56^+^CD3^−^ cells	[32]

Melanoma (primary/metastases)		Low CD56^+^NKG2D^+^ NK cells	[38]

Melanoma (metastases)		Low CD56^+^ NK cells	[21]

Melanoma (nodal/skin metastases)		CD56^+^ NK cells rarely present in melanoma	[39]

Melanoma (nodal metastases)	NKp30, NKG2D expression inversely correlated with number of tumor cells in the LN	NK cells surround tumor cell cluster	[40]

Melanoma (nodal metastases)		Enrichment of CD56^dim^ KIR^+^CD57^+^ cytotoxic NK cells	[41]

Colorectal cancer		Scarce NKp46^+^ infiltrating NK cells (despite high levels of chemokines)	[42]

Colorectal cancer	Reduced NKp46, NKp30, DNAM-1 expression		[43]

Colorectal cancer (lung metastases)		Low NKp46^+^ NK cell infiltrate	[44]

Breast cancer	Expression/function of NKp30, NKG2D in infiltrating NK cells decreases with disease progression		[45]

Breast cancer	Enrichment of CD56^bright^ Perf^low^ poorly cytotoxic NK cells		[35]

Renal cell carcinoma (lung metastases)		High NKp46^+^ NK cell infiltrate correlates with improved survival	[44]

GIST (GastroIntestinal Stromal Tumors)		Substantial NKp46^+^ NK cell infiltrate mainly surrounding tumor nests	[46]

GIST (GastroIntestinal Stromal Tumors)		Low NK cell infiltration/high metastases at diagnosis	[46]

GIST (GastroIntestinal Stromal Tumors)		High NK cell infiltration/prolonged progression-free survival after imatinib treatment	[47]
